# Genome-Wide Association and RNA-Seq Analyses Reveal a Potential Candidate Gene Related to Oil Content in Soybean Seeds

**DOI:** 10.3390/ijms25158134

**Published:** 2024-07-25

**Authors:** Hongchang Jia, Dezhi Han, Xiaofei Yan, Lei Zhang, Jili Liang, Wencheng Lu

**Affiliations:** Heihe Branch of Heilongjiang Academy of Agricultural Sciences, Heihe 164300, China; jiahongchang@haas.cn (H.J.); handezhi2008@163.com (D.H.); woque3@163.com (X.Y.); hhnkszl@163.com (L.Z.); ljllym1113@163.com (J.L.)

**Keywords:** soybean, genome-wide association study, transcriptome, oil content

## Abstract

Soybean is a crucial crop globally, serving as a significant source of unsaturated fatty acids and protein in the human diet. However, further enhancements are required for the related genes that regulate soybean oil synthesis. In this study, 155 soybean germplasms were cultivated under three different environmental conditions, followed by phenotypic identification and genome-wide association analysis using simplified sequencing data. Genome-wide association analysis was performed using SLAF-seq data. A total of 36 QTLs were significantly associated with oil content (−log10(*p*) > 3). Out of the 36 QTLs associated with oil content, 27 exhibited genetic overlap with previously reported QTLs related to oil traits. Further transcriptome sequencing was performed on extreme high–low oil soybean varieties. Combined with transcriptome expression data, 22 candidate genes were identified (|log2FC| ≥ 3). Further haplotype analysis of the potential candidate genes showed that three potential candidate genes had excellent haplotypes, including *Glyma.03G186200*, *Glyma.09G099500*, and *Glyma.18G248900*. The identified loci harboring beneficial alleles and candidate genes likely contribute significantly to the molecular network’s underlying marker-assisted selection (MAS) and oil content.

## 1. Introduction

Lipids are essential metabolic compounds in plants that play a pivotal role in plant growth [1]. The oil content of seeds in different crops exhibits significant variation, with those in maize ranging from 3% to 7%, soybeans ranging from 16% to 23%, rapeseed ranging from 36% to 47%, and peanuts ranging from 40% to 50% [2,3,4,5]. Triacylglycerol serves as the predominant constituent of seed oil, comprising three molecules of long-chain fatty acids and one molecule of glycerol [6].

Soybean, a primary oil crop, synthesizes oil mainly in the endoplasmic reticulum. Using acetyl-CoA and glycerol-3-phosphate (G3P) as substrates for a series of reactions, this process is called the Kennedy pathway [7]. Glycerol-3-phosphate acyltransferases and lysophosphatidic acid acyltransferase catalyze the continuous acylation of the sn-1 and sn-2 sites of G3P, resulting in the formation of phosphatidic acid [8]. Moreover, phosphatidic acid (PA) undergoes dephosphorylation at the sn-3 position, catalyzed by phosphatidic acid phosphohydrolase (PAP), resulting in the formation of diacylglycerol (DAG) [9]. The generated DAG is further acylated to TAG at the sn-3 position, catalyzed by diacylglycerol acyltransferase (DGAT) [10]. Due to its instability in the endoplasmic reticulum, the TAG eventually undergoes sequestration within the seed as an oil body through interaction with specific oil body proteins [11]. It was reported that diacylglycerol acyltransferase (DGAT1-2) plays a pivotal role as a key enzyme in catalyzing oil synthesis, thereby significantly enhancing the oil content in maize seeds [12].

With the advancement of omics technology, high-throughput transcriptome sequencing and genome-wide association analysis have become ubiquitous tools for investigating specific traits. Research reports that a total of 96,432 SNPs were identified with 203 soybean accessions. A total of 44 QTLs were identified to be significantly associated with protein, oil, and amino acid content, and the genes *Glyma.11G015500* and *Glyma.20G050300* were identified as novel candidates for protein and oil content, respectively [13]. The oil content of 588 rapeseed materials was associated with 385,692 SNPs through genome-wide association analysis, resulting in the identification of 17 significant association sites; 13 SNPs were found to be located on chromosomes A3 (11 loci) and A1 (one locus), while 5 novel SNPs were discovered on chromosomes C5 (one locus) and C7 (four loci) [14]. A genome-wide association analysis was performed on grain weight using 24,180 SNPs from 185 soybean materials, resulting in the identification of 34 SNPs significantly correlated with grain weight, including 19 newly discovered QTNs [15]. Two varieties representing different contents of unsaturated fatty acids were selected from 314 soybean materials, and RNA-seq analysis was performed at three different developmental stages for these selected varieties. A total of 2080, 11,343, and 2230 DEGs were identified [16]. Transcriptome analysis of seed embryos at 0 h, 12 h, and 48 h of imbibition revealed that brassinosteroids (BRs) can enhance seed germination by facilitating phosphorylation-mediated reactions to abscisic acid (ABA), while epigenetic modifications may serve as a crucial regulatory mechanism governing seed dormancy and germination [17].

To further elucidate the regulatory mechanism underlying soybean oil content, in this study, oil content of 155 soybean materials was determined, and genome-wide association analysis was performed. Furthermore, extreme high–low oil soybean materials were screened for transcriptome sequencing. The putative genes governing oil content were identified through the integration of GWAS and transcriptome analysis.

## 2. Results

### 2.1. Statistical Analysis of Oil Content

**Figure 1:**
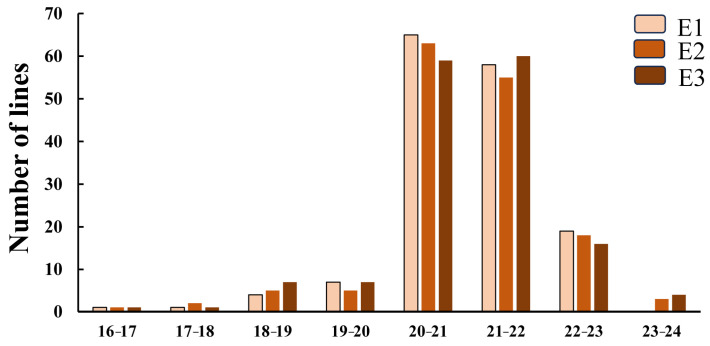
Frequency distribution of oil content in natural population. Note: E1: Nenjiang, E2: Beian, E3: five connected lakes.

A total of 155 soybean materials were utilized in this study, which were collected from three distinct locations in Heilongjiang province, namely, Nenjiang (124°44′ N, 48°42′ E), Beian (47°35′ N, 126°16′ E), and the five connected lakes area (48°18′ N, 126°04′ E) in 2023. The oil content of the 155 soybean materials exhibited regional variations, with the coefficient of variation ranging between 4.4% and 4.6% across different regions (Table S1). The phenotypic changes observed in Nenjiang, Beian, and the five connected lakes area ranged from 16.9% to 22.5%, 16.4% to 22.4%, and 16.6% to 22.3%, respectively (Figure 1).

### 2.2. Population Structure Analysis

In this study, we performed specific-locus amplified fragment sequencing on 155 soybean samples, and a total of 23,131 high-quality SNPs were obtained (MAF > 0.05, missing data < 10%). The SNPs obtained above were evenly distributed across the 20 chromosomes of soybean (Figure S1A). Principal component analysis (PCA) showed that there was an inflection point at PC3 (Figure S1B). The population exhibited no discernible stratification, while the first three principal components exerted a significant influence on the population structure (Figure S1C). The genetic relationships among 155 soybean germplasm were determined based on the SNP genotyping results (Figure S1D).

### 2.3. Genome-Wide Association Study

**Figure 2:**
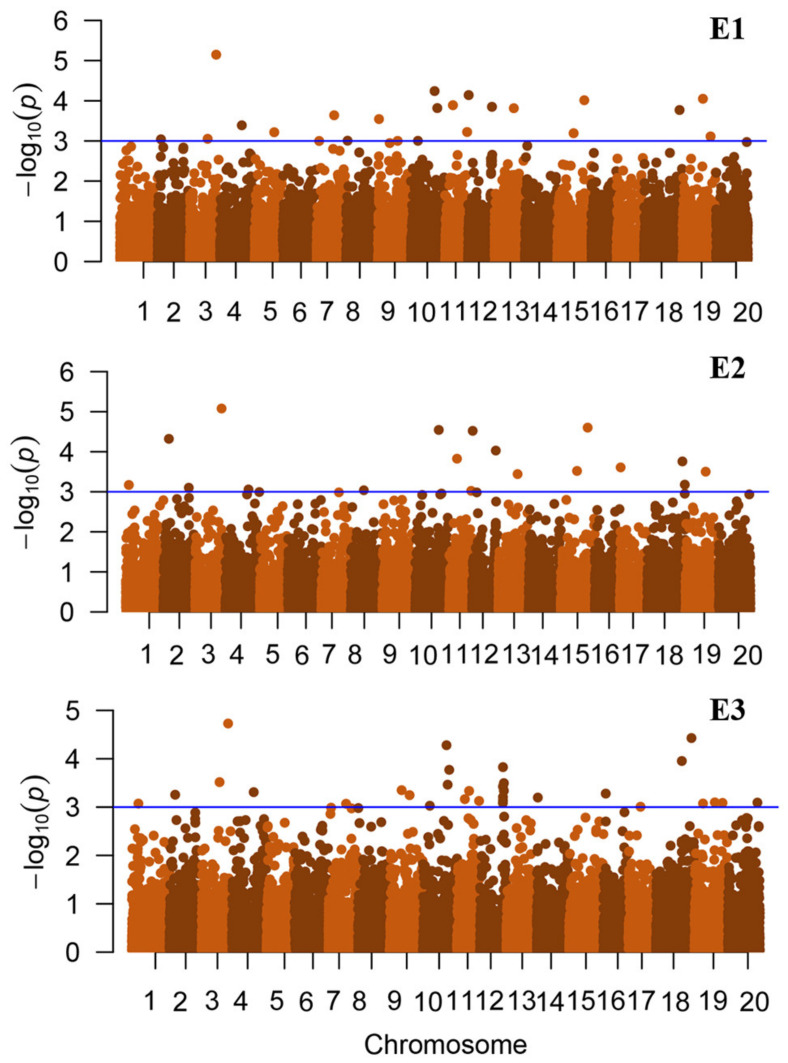
The genome-wide association analysis of oil content across three locations: E1: Nenjiang, E2: Beian, and E3: five connected lakes.

We analyzed the oil content in three environments using a compressed mixed linear model (CMLM) with 23,131 SNP markers across the genome through a genome-wide association study. The results showed that 36 QTLs were significantly associated with oil content (−log10(*p*) > 3) (Figure 2). The subsequent analysis revealed that stable QTLs that could be screened under multiple environmental conditions were mainly distributed on chromosomes 3, 4, 7, 9, 10, 11, 12, 13, 15, 18, and 19. Out of the 36 QTLs associated with oil content, 27 exhibited genetic overlap with previously reported QTLs related to oil traits (Table 1).

### 2.4. Functional Prediction of Candidate Genes by GWAS and RNA-Seq Analysis

**Figure 3:**
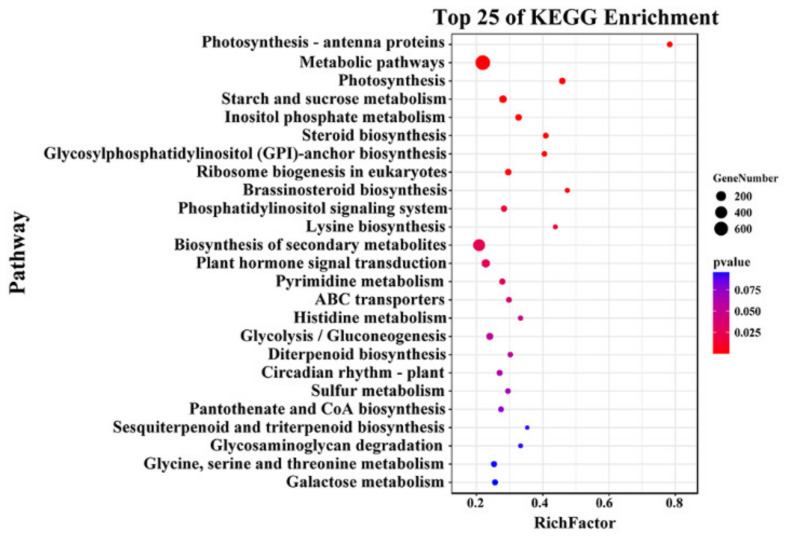
KEGG enrichment of DEGs.

To further identify candidate genes regulating oil content, this study conducted transcriptome sequencing on the soybean materials with extremely high and low oil contents. The results showed that 7774 upregulated and 1801 downregulated differentially expressed genes (DEGs) were identified (|log2FC| ≥ 1) (Figure S2). The obtained differential genes were subjected to KEGG enrichment analysis, revealing that a total of 1686 differential genes were significantly enriched in KEGG pathways. The DEGs were predominantly enriched in metabolic pathways (ko01100, *p* < 8.83 × 10^−8^), starch and sucrose metabolism (ko00500, *p* < 0.0005), photosynthesis-antenna proteins (ko00196, *p* < 1.52 × 10^−9^), and photosynthesis (ko00195, *p* < 1.45 × 10^−7^) (Figure 3).

This study explored novel candidate genes regulating oil content, identifying a total of 706 candidate genes from the 100 kb range surrounding the peak SNP (QTL) (Table S2). Combined with transcriptome expression data, 22 candidate genes were differentially expressed (|log2FC| ≥ 3) (Table 2). According to the functional annotation of the candidate gene, *Glyma.20G208400* exhibits homology with Arabidopsis *AT1G62510*. It was reported that overexpression of the aspartate aminotransferase gene can increase the amino acid content of seeds in rice [33]. The gene *Glyma.03G186200* exhibits homology with Arabidopsis *AT5G03530*. Previous studies have demonstrated the role of the small GTPase ARL8B gene in mediating lipid droplet transformation [34]. The gene *Glyma.17G016900* exhibits homology with the Arabidopsis gene *AT3G06140*. It was found that A U-box type E3 ubiquitin ligase plays a regulatory role in lipid accumulation [35]. The above genes were found to have higher expression levels. In addition, other identified genes based on expression levels were also defined as candidate genes. Additionally, four differential genes were selected for real-time PCR, and the results showed that the results of qRT-PCR were basically consistent with the transcriptome data (Figure S3).

### 2.5. Gene-Based Association and Haplotype Analysis of Candidate Genes

**Figure 4:**
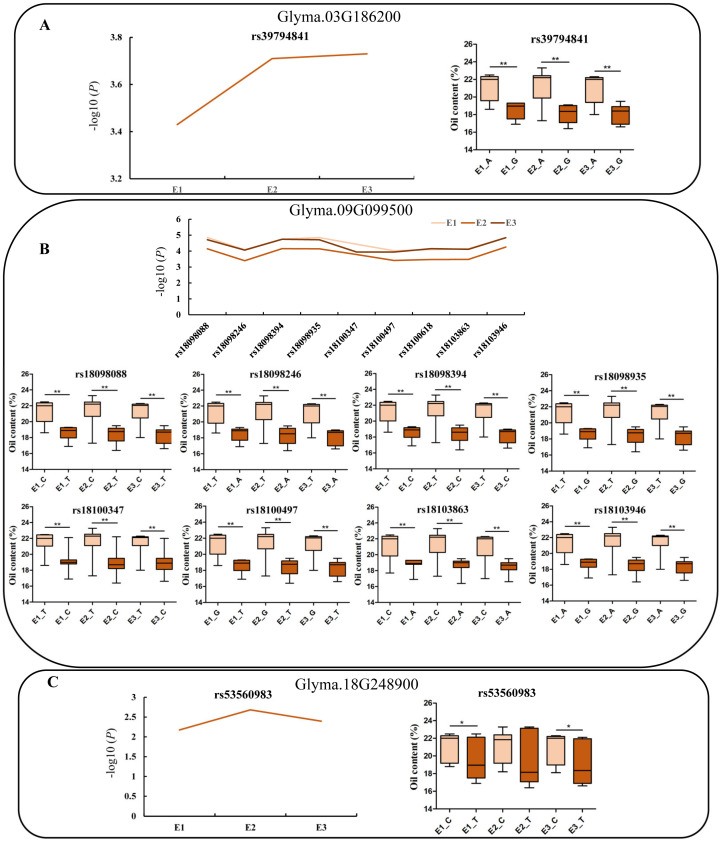
Haplotypes analysis of genes with variations related to oil content. Note: E1: Nenjiang, E2: Beian, E3: five connected lakes. * and ** indicates significance at *p* < 0.05 and *p* < 0.01.

In order to determine the sequence variation in candidate genes, association analysis was conducted using the SNPs of the candidate genes and phenotypic traits through GLM. Based on the association analysis, one SNP was identified in the UTR region in the *Glyma.03G186200* gene (−log10(*p*) > 2.5), where the oil content of the A allele was significantly higher than that of the G allele (Figure 4A). A total of eight single-nucleotide polymorphisms (SNPs) were identified in the upstream, UTR5, exonic, and UTR3 regions of the *Glyma.09G099500* gene across the three environments (−log10(*p*) > 2.5). The oil content associated with the C/T/T/T/T/G/C/A alleles was significantly higher than that of the T/A/C/G/C/T/A/G alleles (Figure 4B). One SNP was identified in the exonic region in the *Glyma.18G248900* gene in the three environments (−log10(*p*) > 2.5), and the oil content of C alleles in environments E1 and E3 was significantly higher than that of T alleles, while there was no significant difference in oil content between the C and T alleles in the E2 environment (Figure 4C).

### 2.6. Co–Expression Analysis of Transcription Factors and Candidate Genes

In this study, transcription factors were collected from the PlantTFDB database, and their differential expression levels were determined through transcriptome data. A total of 720 differentially expressed transcription factors were obtained, which were divided into 52 categories. The most abundant TF families mainly included bHLH, ERF, MYB, NAC, and bZIP (Figure S4).

**Figure 5:**
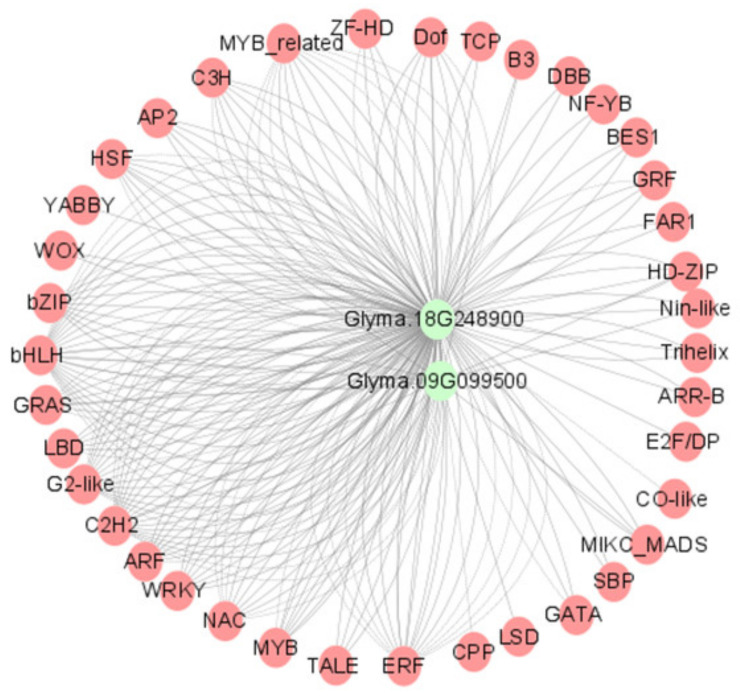
Co-expression network analysis of candidate genes and transcription factors.

Subsequently, the integration of 720 differential transcription factors with three candidate genes generated 230 subnetworks (r > 0.98). *Glyma.18G248900* was found to be significantly positively associated with C2H2 (*Glyma.07G107500*, r > 0.99, *p* < 1.72 × 10^−8^), bZIP (*Glyma.11G183700*, r > 0.99, *p* < 2.43 × 10^−8^), G2-like (*Glyma.13G316600*, r > 0.99, *p* < 3.14 × 10^−8^), bHLH (*Glyma.06G100000*, r > 0.99, *p* < 4.07 × 10^−8^), and ARF (*Glyma.12G071000*, r > 0.99, *p* < 6.11 × 10^−8^). *Glyma.09G099500* was found to be significantly positively associated with HD-ZIP (*Glyma.06G100000*, r > 0.99, *p* < 8.46 × 10^−8^), ERF (*Glyma.06G100000*, r > 0.99, *p* < 9.89 × 10^−8^), and C2H2 (*Glyma.01G134200*, r > 0.99, *p* < 4.17 × 10^−6^) (Figure 5, Table S3).

## 3. Discussion

Lipid synthesis in plants is typically accomplished through multiple pathways and cell types. Although the fatty acid synthesis pathway has been relatively well elucidated, further investigation is still required to understand the genetic factors that regulate lipid metabolism pathways. Genome-wide association studies (GWAS) have emerged as a prominent approach for elucidating the genetic loci associated with agronomic traits in crops [36,37]. Currently, the QTLs associated with significant traits, such as branch number [38], seed size [39], flowering time [40], and stress resistance [41], have been successfully identified. Compared to traditional QTL mapping, GWAS detect more genetic loci due to abundant molecular markers and larger sample sizes.

Researchers have analyzed the genetic basis for regulating soybean oil content. Previous studies have conducted linkage analyses of the protein and oil contents in RIL populations, identifying 19 major QTLs associated with oil content located on chromosomes 1, 2, 3, 6, 8, 10, 11, 13, 16, and 20 [42]. Tian et al. conducted a QTL analysis on the oil content of the FW-RIL population, and a total of 17 QTLs related to oil content were identified [43]. In this study, a total of 36 QTLs were identified to be significantly associated with oil content (−log10(*p*) > 3), which were located on chromosomes 1, 2, 3, 4, 5, 7, 9, 10, 11, 12, 13, 14, 15, 16, 18, 19, and 20, respectively. Furthermore, 27 QTLs were found to exhibit genetic overlap with previously documented QTLs associated with oil traits (Table 1).

The continuous advancement of omics data has revealed that single-omics analysis may introduce bias in the investigation of certain plant traits, and it presents limitations in elucidating plant regulatory mechanisms. Multi-omics technology has been widely used in screening important markers of target traits and mining the related candidate genes. A total of 52 SNPs were identified and found to be correlated with four chlorophyll fluorescence parameters through transcriptome and GWAS’ analysis. Additionally, RNA-seq analysis led to the screening of three candidate genes in important genomic regions [44]. Song et al. used transcriptome and GWAS to analyze soybean seed coat color and found that 182 differentially expressed genes (DEGs) were screened out from five QTLs, including *CHS*, *MYB*, and *F3′H* genes [45]. In this study, a total of 22 potential candidate genes (|log2FC| ≥ 3) were identified based on transcriptome methods and GWAS (Table 2). Haplotype analysis revealed that three candidate genes, *Glyma.03G186200*, *Glyma.09G099500*, and *Glyma.18G248900*, had excellent haplotypes (Figure 4). One SNP was identified in the UTR region in the *Glyma.03G186200* gene (−log10(*p*) > 2.5), where the oil content of the A allele was significantly higher than that of the G allele (Figure 4A). The *Glyma.03G186200* gene encodes the RAB GTPase homolog C2A, and previous studies have demonstrated that the small GTPase ARL8B gene is involved in mediating lipid droplet transformation [34]. RabC1 has been identified as a crucial regulator essential for the modulation of lipid droplet dynamics and lipid metabolism. The findings demonstrate that RabC1 is capable of interacting with SEIPIN2 and SEIPIN3, both localized in the endoplasmic reticulum to modulate the mobilization of lipid droplets and ensure adequate lipid availability [46]. Previous studies demonstrated the pivotal role of RabC1 GTPase in regulating Arabidopsis growth and seed development [47]. A total of eight SNPs were identified in the *Glyma.09G099500* gene (−log10(*p*) > 2.5) across the upstream, UTR5, exonic, and UTR3 regions. Among these variants, the oil content of the C/T/T/T/T/G/C/A alleles was significantly higher than that of the T/A/C/G/C/T/A/G alleles (Figure 4B). The *Glyma.09G099500* gene encodes a cation efflux family protein. The expression level of the MTP8 gene in Arabidopsis exhibits a continuous increase during seed development [48]. Furthermore, one SNP was detected in the exonic region of the *Glyma.18G248900* gene across the three environments (−log10(*p*) > 2.5). The oil content associated with allele C in environments E1 and E3 was significantly higher than that associated with allele T, while no significant difference in oil content was observed between alleles C and T in environment E2 (Figure 4C). The *Glyma.18G248900* gene encodes an unknown protein. Furthermore, it was observed that Glyma-18G248900 exhibits a significant positive association with *bZIP*, *C3H*, and *Dof*. Overexpression of the soybean *bZIP* transcription factor (*GmbZIP123*) leads to an increase in oil content in transgenic Arabidopsis seeds [49]. Previous studies demonstrated that the overexpression of *GhDof1* leads to an increase in the oil content of upland cotton [50]. This study postulates that the regulation of the *Glyma18G248900* gene and the subsequent changes in soybean oil content may be mediated by *bZIP* and *Dof* transcription factors. Meanwhile, it was found that *Glyma.09G099500* is significantly positively correlated with *bHLH*, *MYB*, and *GRAS*. There have been reports suggesting that MYB1 plays a crucial role in the induction of *FAT1* expression and facilitates the efficient transport of fatty acids from chloroplasts, which represents a pivotal step in lipid biosynthesis within the endoplasmic reticulum [51].

## 4. Materials and Methods

### 4.1. Plant Materials

In this study, 155 soybean germplasms were used as experimental materials (Table S4). All materials were planted in three designated test locations, namely, Nenjiang (124°44′ N, 48°42′ E), Beian (47°35′ N, 126°16′ E), and the five connected lakes area (48°18′ N, 126°04′ E). Field germplasm was planted with a row length of 2 m, a spacing between rows of 0.6 m, and a density of 30 plants per row. Fifteen soybean plants at the mature stage were randomly selected for the determination of oil content. An Infratec 1241 NIR grain analyzer (FOSS, Hoganas, Sweden) was utilized for quantifying the soybean oil content.

### 4.2. Germplasm Population Genotype Analysis

The genomic DNA of each leaf sample was extracted using the CTAB method. The specific-locus amplified fragment sequencing (SLAF-seq) technique was employed for the detection of amplified fragment sequencing in 155 soybean germplasms. The test samples were subjected to digestion using restriction endonucleases (*Mse* I and *Hae* III), resulting in the generation of fragments ranging from 300 bp to 500 bp in length. The barcode method and an Illumina Genome Analyzer II System (Illumina Inc., San Diego, CA, USA) were utilized to generate 45 bp sequence reads at both ends of the sequencing tags from each accession library. The alignment of the acquired raw paired-end reads to the reference genome (Glycine max Wm82. a2. v1) was conducted utilizing BWA software (Version: 0.6.1-r104). For subsequent association analysis, a total of 23,131 SNPs with a minimum allele frequency (MAF) ≥ 5% and deletion rate ≤ 10% were selected using GATK (version 2.4-7-g5e89f01) and SAMtools (Version: 0.1.18).

### 4.3. Population Structure Evaluation, Linkage Disequilibrium, and Genome-Wide Association Analysis

The oil content of 155 soybean materials was analyzed using a compressed mixed linear model (CMLM) in the GAPIT package, employing 23,131 single-nucleotide polymorphisms (SNPs). Significant SNP loci were screened with −log10(*p*) > 3 as the threshold. The first three principal component analyses (PCAs) were included as covariates in the subsequent analysis.

### 4.4. Transcriptome Sequencing Analysis

Total RNA extraction from soybean grain at the R6 stage was performed using TRIzol reagent (Invitrogen, Carlsbad, CA, USA). The eukaryotic mRNA was selectively enriched using magnetic beads conjugated with Oligo (dT) following the assessment of total RNA quality in the samples. Double-stranded cDNA was synthesized using mRNA as a template and six-base random primers, followed by purification and end repair of the cDNA. The size selection of cDNA fragments and accurate quantification of the effective concentration for library inspection were achieved using AMPure XP beads. The sequencing was conducted using the IlluminaHiSeq platform after meeting the qualification criteria. The raw reads obtained through sequencing must undergo quality control (QC) to eliminate low-quality reads and bases, resulting in the acquisition of high-quality clean reads. The soybean reference genome (glycine max Wm82. a2. v1) was utilized for sequence alignment. The differentially expressed genes were identified using thresholds of |log2Fold Change| > 1 and *p* < 0.05. Three biological replicates of each material were applied in this study.

### 4.5. Prediction of Candidate Genes

The 100 kb genomic region (upstream and downstream) of each significant SNP is defined as the putative candidate gene. The identification of putative candidate genes within the confidence interval was achieved by employing a combination of GWAS and transcriptome analysis, aiming to explore the regulatory mechanisms underlying oil content variation. A set of 28 soybean lines (14 extremely high oil and 14 extremely low oil) was derived from genome resequencing data to discern variations in candidate genes encompassing 5′UTRs, 3′UTRs, exons, and promoter regions. A general linear model (GLM) implemented in TASSEL 5.0 software was employed for haplotype analysis of candidate genes.

### 4.6. Co-Expression Analysis

The online plantTFDB database was utilized for the screening of soybean transcription factors. The differentially expressed transcription factors were selected for correlation analysis with potential candidate genes, and those with a Pearson correlation coefficient threshold (r > 0.98, *p* < 0.05) were selected. The visualization of the co-expression network was generated using Cytoscape 3.10.1 software.

### 4.7. Quantitative Real-Time PCR

Differential candidate genes were screened and analyzed by quantitative real-time PCR. The quantitative real-time PCR was conducted using a SYBR Green Realtime PCR Master Mix Kit (TOYOBO, Osaka, Japan). The relative expression level was determined using the 2^−ΔΔct^ method. Three biological replicates and three technical replicates were applied in this study. GmACTIN4 was used as internal control. All primers of qRT-PCR were generated in Table S5.

## 5. Conclusions

In this study, a total of 155 soybean materials were utilized. A total of 36 QTLs were found to be significantly correlated with oil content by GWAS analysis. Through the integrated GWAS and transcriptome analysis, 22 potential candidate genes were identified in this study. Haplotype analysis revealed that three candidate genes, *Glyma.03G186200*, *Glyma.09G099500*, and *Glyma.18G248900*, had excellent haplotypes. Furthermore, co-expression analysis revealed a significant correlation between *Glyma.09G099500* and *Glyma.18G248900* with *bHLH*, *bZIP*, *MYB*, and *Dof* transcription factors.

## Figures and Tables

**Table 1:** Genetic overlap between the selected QTLs and known QTLs.

Env	Locus Name	Chr.	Pos	−Log10(*p*)	Known QTLs
E2	rs4477127	1	4,477,127	3.17	[18]
E3	rs9691814	1	9,691,814	3.07	[18]
E2	rs38563127	2	38,563,127	3.1	
E1	rs4964805	2	4,964,805	3.04	
E3/E2	rs8406199	2	8,406,199	3.26/4.32	[19]
E3/E1	rs26910547	3	26,910,547	3.51/3.05	[20]
E3/E2/E1	rs39790117	3	39,790,117	4.73/5.08/5.15	[21]
E3/E1	rs32815793	4	32,815,793	3.31/3.39	
E2	rs35005741	4	35,005,741	3.06	
E1	rs29415473	5	29,415,473	3.22	
E3/E1	rs26521346	7	26,521,346	3.07/3.64	
E2	rs19738745	8	19,738,745	3.04	
E1	rs2147095	8	2,147,095	3.01	[22]
E3	rs18054299	9	18,054,299	3.35	[23]
E1	rs1843998	9	1,843,998	3.54	[23,24]
E3/E1	rs29978290	9	29,978,290	3.25/3	[23]
E3/E1	rs10499458	10	10,499,458	3.03/3	[25]
E3/E2/E1	rs35631359	10	35,631,359	4.28/4.55/4.24	[23]
E3	rs37347397	10	37,347,397	3.46	[23]
E3/E1	rs39714349	10	39,714,349	3.77/3.82	[23]
E3/E2/E1	rs11758049	11	11,758,049	3.16/3.83/3.89	[26]
E3	rs18218600	11	18,218,600	3.33	[26]
E3/E2/E1	rs33193693	11	33,193,693	3.13/3.02/3.22	[25]
E3	rs34470363	12	34,470,363	3.08	[27]
E3	rs34585918	12	34,585,918	3.43	[27]
E3	rs34600639	12	34,600,639	3.14	[27]
E3	rs34601911	12	34,601,911	3.21	[27]
E3	rs34654632	12	34,654,632	3.83	[27]
E3/E2/E1	rs35979450	12	35,979,450	3.34/4.03/3.85	[28,29]
E3	rs36025778	12	36,025,778	3.5	[28,29]
E2/E1	rs917378	12	917,378	4.52/4.14	
E2/E1	rs28963005	13	28,963,005	3.44/3.81	[28,30]
E3	rs1731411	14	1,731,411	3.2	
E2/E1	rs24670157	15	24,670,157	3.52/3.19	[29,31]
E2/E1	rs40773008	15	40,773,008	4.6/4.01	[32]
E3	rs3534128	16	3,534,128	3.28	[19]

Note: E1: Nenjiang, E2: Beian, E3: five connected lakes.

**Table 2:** Candidate differential genes were identified by transcriptome and GWAS.

Env	SNP	Gene ID	Log2FC	Arabidopsis	Description
E3	rs29978290	*Glyma.09G123900*	3.11	/	/
E3	rs18218600	*Glyma.11G170100*	3.16	AT1G01040	Dicer-like 1
E3	rs39790117	*Glyma.03G186200*	3.19	AT5G03530	RAB GTPase homolog C2A
E2	rs19738745	*Glyma.08G235400*	3.23	AT1G73260	Kunitz trypsin inhibitor 1
E3	rs35979450	*Glyma.12G198700*	3.25	AT5G62990	Ubiquitin carboxyl-terminal hydrolase family protein
E1	rs1843998	*Glyma.09G022000*	3.3	AT3G54950	Patatin-like protein 6
E3	rs37347397	*Glyma.10G139700*	3.72	AT3G62020	Germin-like protein 10
E3	rs53468862	*Glyma.18G246800*	3.96	AT3G25500	Formin homology 1
E3	rs35979450	*Glyma.12G199300*	4.06	AT4G03270	Cyclin D6
E3	rs29978290	*Glyma.09G124200*	4.21	AT1G70670	Caleosin-related family protein
E3	rs53468862	*Glyma.18G248900*	5	/	/
E2	rs24670157	*Glyma.15G201500*	5.12	/	/
E3	rs3534128	*Glyma.16G037600*	5.4	AT5G39890	Protein of unknown function
E2	rs4477127	*Glyma.01G040600*	8.12	AT1G10350	DNAJ heat shock family protein
E2	rs57089675	*Glyma.18G293300*	8.35	AT2G04090	MATE efflux family protein
E2	rs1251586	*Glyma.17G016900*	8.7	AT3G06140	RING/U-box superfamily protein
E1	rs4964805	*Glyma.02G054400*	9.04	AT5G66440	/
E2	rs24670157	*Glyma.15G201000*	−7.71	AT1G21280	/
E3	rs44446658	*Glyma.20G208400*	−6.94	AT1G62510	Bifunctional inhibitor/lipid-transfer protein/seed storage 2S albumin superfamily protein
E3	rs33193693	*Glyma.11G238300*	−3.64	AT2G22250	Aspartate aminotransferase
E3	rs18054299	*Glyma.09G099500*	−3.54	AT1G16310	Cation efflux family protein
E3	rs18196571	*Glyma.17G174700*	−3.07	AT5G09910	Ras-related small GTP-binding family protein

Note: E1: Nenjiang, E2: Beian, E3: five connected lakes.

## Data Availability

Data are contained within the article or Supplementary Materials.

## Supplementary Materials

The supporting information can be downloaded at: https://www.mdpi.com/article/10.3390/ijms25158134/s1.
