# Supplementary material for: Genome-Wide Association and RNA-Seq Analyses Reveal a Potential Candidate Gene Related to Oil Content in Soybean Seeds
Source: Int J Mol Sci. 2024 Jul 25;25(15):8134. doi: 10.3390/ijms25158134 (PMC11311756; doi:10.3390/ijms25158134)
Supplement: Supplementary file 1 [file ijms-25-08134-s001.zip › TableS1 and Figures S1-S4.pdf]

Table S1. Statistical analysis of oil content in soybean

Location	Min (%)	Max (%)	Mean (%)	SD	CV (%)
E1	16.9	22.5	20.93	0.92	4.4
E2	16.4	22.6	20.88	1.02	4.9
E3	16.6	22.3	20.87	0.97	4.6

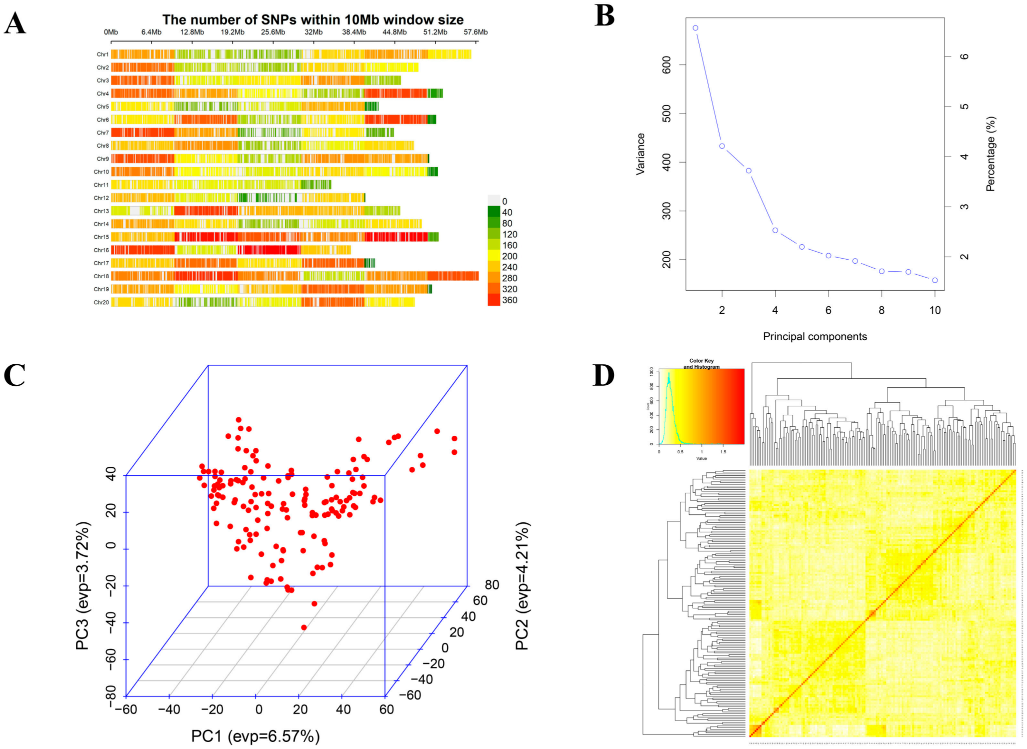

Figure S1. Genetic features of the mapping population. (A). The density distribution of SNPs. (B). The first three principal components reflected by SNPs used in the GWAS. (C). Population structure of soybean germplasm. (D). A heatmap of the kinship matrix of the 155 soybean accessions.

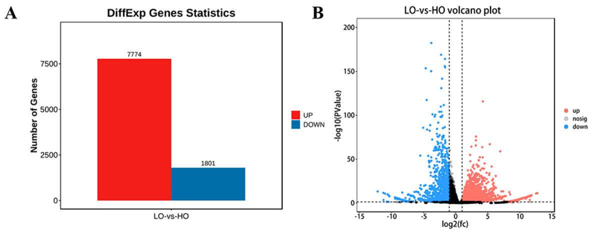

Figure S2. Gene expression level analysis.

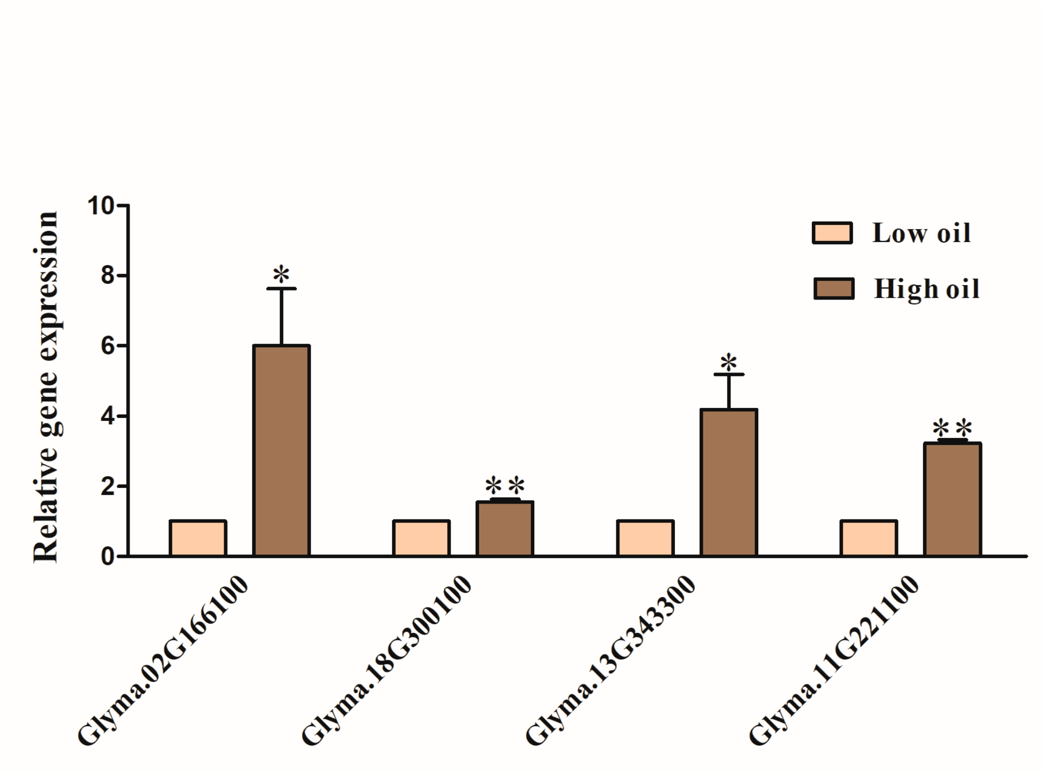

Figure S3. qRT-PCR analysis of candidate genes. * and ** indicates significance at $p < 0.05$ and $p < 0.01$.

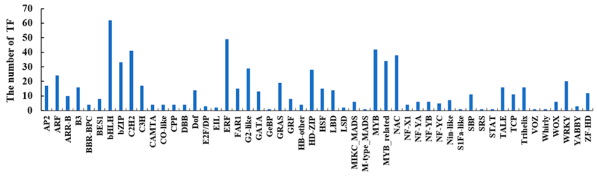

Figure S4. The number of TFs.
